# Association between serum vitamin D and uric acid in the eastern Chinese population: a population-based cross-sectional study

**DOI:** 10.1186/s12902-020-00560-1

**Published:** 2020-06-03

**Authors:** Yingchao Chen, Jing Cheng, Yi Chen, Ningjian Wang, Fangzhen Xia, Chi Chen, Bing Han, Yingli Lu

**Affiliations:** Institute and department of Endocrinology and Metabolism, Shanghai Ninth People’s Hospital Affiliated to Shanghai Jiaotong University School of Medicine, No.639 Zhizaoju Road, Shanghai, China

**Keywords:** Uric acid, Vitamin D, Hyperuricemia, Public health

## Abstract

**Background:**

Uric acid (UA) is the end product of purine metabolism, which is thought to be related to many human diseases, such as nephrolithiasis, gout, cardiovascular disease (CVD), type 2 diabetes mellitus, metabolic syndrome. However, the relationship between serum UA (SUA) and 25(OH) D is still unclear in the eastern Chinese population.

**Methods:**

We did a population-based observational investigation, which included 12,770 residents living in eastern China. Ultimately, data from 9220 subjects were analyzed. Serum 25(OH) D, SUA, fasting plasma glucose (FPG), fasting insulin, HbA1c and other metabolic parameters were tested. Waist circumference (WC), weight and height were also measured. Questionnaires were collected from these subjects for information on smoking and drinking status.

**Results:**

We enrolled 9220 Chinese adults, including 3681 males (age 55.57 ± 13.23 years) and 5539 females (age 54.31 ± 12.83 years). The levels of SUA were 352.07 ± 79.25 nmol/L and 269.29 ± 64.68 nmol/L in males and females, respectively. The proportion of adults with hyperuricemia (HUA) was 12.26% in the total population. Levels of SUA were positively associated with 25(OH) D, and the incidence of HUA increased 9.4% for every 10 nmol/L increase in 25(OH) D (*P* < 0.001).

**Conclusions:**

SUA was positively associated with 25(OH) D in the eastern Chinese population. Higher levels of serum 25(OH) D may be a potential predictor of HUA.

## Background

Vitamin D is an essential fat-soluble vitamin for humans. It can be produced in the skin when the epidermis is exposed to ultraviolet B radiation or it can be obtained from the diet, including ergocalciferol (D2) from plants and cholecalciferol (D3) mostly from marine life. The primary source of vitamin D is the keratinocytes of the skin [1]. Vitamin D is activated by hydroxylases, namely, forms of cytochrome P450, to become the active hormone (1,25(OH)_2_D). As 25(OH) D has a significantly longer half-life than 1,25(OH)_2_D, the serum level of 25(OH) D is considered to be the most stable and reliable indicator of vitamin D status.

Vitamin D is generally regarded as an important pro-hormone, that can participate in regulating the metabolism of calcium phosphate and bone physiology. Recently, an increasing number of studies have shown that vitamin D is also involved in many other diseases. Through its ubiquitously expressed receptor, calcitriol displays potent anti-angiogenic and anti-inflammatory activity [2]. The active vitamin D metabolite can alter DNA transcription through vitamin D receptors (VDRs), heterodimerized with retinoic X receptors, which bind to the regulatory sites in target genes [2].

Serum uric acid (SUA) is the end product of purine metabolism in humans. Most circulating uric acid (UA) is freely filtered in the kidneys, which excrete approximately 60–70% of the total UA from the body [3]. More than 30 years ago, Ames et al. hypothesized that higher SUA levels might have been beneficial during hominoid evolution because of the antioxidant properties of UA [4]. On the other hand, UA in excess may cause nephrolithiasis and gout. And it has been proposed to be linked to many other human diseases [5]. UA is an independent risk factor for insulin resistance, cardiovascular disease (CVD), nonalcoholic fatty liver disease (NAFLD) [6, 7], type 2 diabetes mellitus, metabolic syndrome, and atherosclerosis [8, 9]. Impaired renal function may increase SUA concentration by decreasing renal excretion [10]. Reduced nephron mass and/or 1-α hydroxylase enzyme activity has been shown to be associated with a decline in 1,25(OH)_2_D levels in patients with chronic kidney disease (CKD) [11]. Previous studies have found that vitamin D deficiency was a predictor of CKD [12].

Vitamin D insufficiency was found significantly associated with elevated UA among postmenopausal Chinese Han women [13]. Another study showed that lower concentrations of SUA predict lower vitamin D levels in patients with type 2 diabetes and CKD [14]. However, there were few studies focused on vitamin D and UA in the general population. In this study, we intended to investigate the relationship between 25(OH) D and SUA in the general population. Our research will extend our understanding of the relationship between vitamin D and SUA.

## Methods

### Study population

**Fig. 1:**
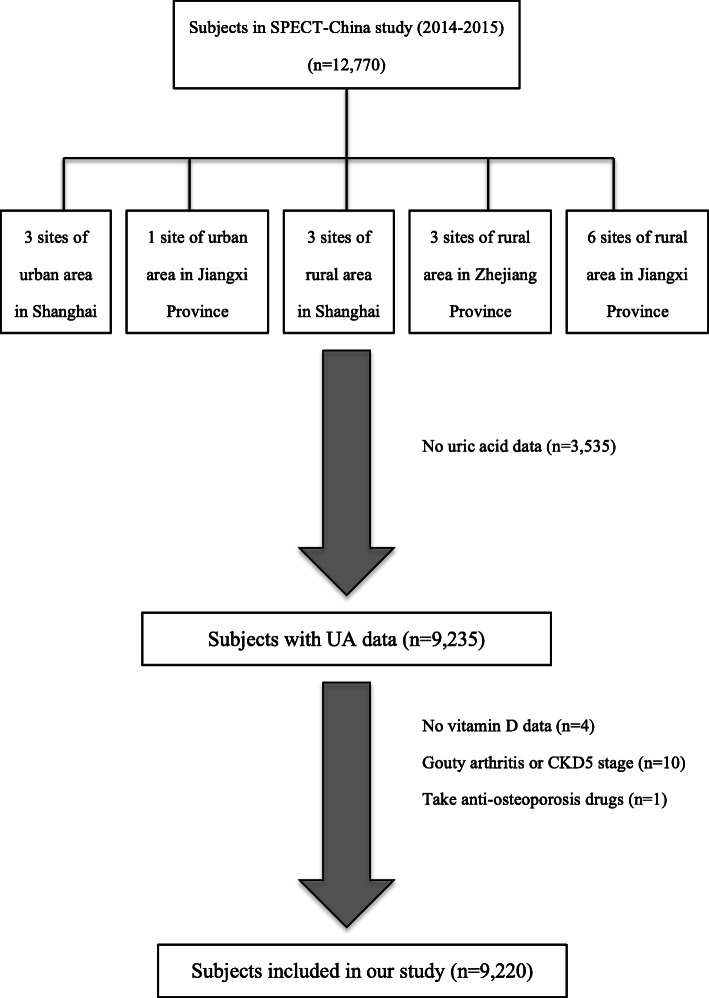
Flowchart of this study. We totally collected 12,770 subjects. After excluding participants who had missing data or specific disease states, finally, 9220 subjects were included

The Survey on Prevalence in East China for Metabolic Diseases and Risk Factors, 2014 (SPECT-China, 2014) is a population-based cross-sectional survey on the prevalence of metabolic diseases and risk factors in eastern China. The registration number is ChiCTR-ECS-14005052_(www.chictr.org). In this study, 12,770 residents from 22 sites in Shanghai Municipality, Zhejiang Province, Jiangsu Province, Anhui Province and Jiangxi Province were enrolled from January 2014 to December 2015. Chinese citizens ≥18 years old who had lived in their current area for ≥6 months were selected. We excluded subjects with severe communication problems, acute illness or who were unwilling to participate. We also excluded those who had no UA data (*n* = 3, 535), no vitamin D data (*n* = 4), and gouty arthritis or stage 5 CKD (*n* = 10), as well as those who took anti-osteoporosis drugs (*n* = 1). Ultimately, 9220 subjects were included (Fig. 1). This study was approved by the ethics committee of Shanghai Ninth People’s Hospital affiliated with Shanghai Jiaotong University School of Medicine. Written consent was obtained from all the participants.

### Measurements and definition

The homeostasis model assessment of insulin resistance (HOMA-IR) was performed by means of fasting glucose (mmol/L) × fasting insulin (mIU/L)/22.5. Weight and height were measured with participants wearing light clothing and no shoes. Body mass index (BMI) was calculated as weight (Kg)/height squared(m^2^). Systolic blood pressure (SBP), diastolic blood pressure (DBP) and heart rate were measured three times with a sphygmomanometer (TERUMO-Elemano). The mean of the three records was used in the analysis. Waist circumference (WC) was measured at the level of 1 cm above the umbilicus. Hyperuricemia (HUA) was defined by UA > 420 μmol/L in men and > 360 μmol/L in women. Demographic information and lifestyle risk factors were gathered from standard questionnaires by trained stuff. Drinking and smoking status was divided into never drinking/smoking and past or current drinking/smoking.

### Assessment of biomarkers

Blood samples were obtained after fasting for at least 8 h; the samples were immediately centrifuged (2000 rpm for 15 min) at room temperature, and stored at − 20 °C when collected and shipped by air in dry ice within 2–4 h of collection to a central laboratory certified by the College of American Pathologists. All plasma and serum samples were frozen at − 80 °C after laboratory testing. Biochemical indexes, including the fasting plasma glucose (FPG), total cholesterol (TC), triglycerides (TG), low-density lipoprotein (LDL), alanine aminotransferase (ALT) were analyzed by Beckman Coulter AU680 (Bera, USA). Serum 25(OH) D (SIEMENS ADVIA Centaur XP, Siemens, Germany) and insulin (Abbott i2000 SR, Chicago, USA) were measured using the chemiluminescence method. Glycated hemoglobin (HbA1c) was detected using high-performance liquid chromatography (HPLC) with MQ-2000PT (Medconn Technology, Shanghai, China) using a commercial reagent (HuaChen biological reagent co., LTD, Shanghai, China).

### Statistical analysis

Data analyses were performed using IBM SPSS Statistics, Version 22 (IBM Corporation, Armonk, New York). All *P* values were two-sided. *P <* 0.05 was considered statistically significant. Continuous variables were summarized as mean ± standard deviation (SD). Categorical variables were expressed as a percentage (%). UA was divided into quartiles. To compare the differences among quartiles, one-way ANOVA was used for continuous variables. Pearson’s χ2 test was used for categorical variables.

Pearson’s correlation was used to analyze the relationship between uric acid and each potentially associated factor, including 25(OH) D, blood glucose, blood pressure, lipids, BMI, WC and ALT. Linear regression analysis was conducted to investigate the independent associations of UA with 25(OH)D. Logistic analysis was performed to investigate the increased risk of HUA for every 10-nmol/L increase in 25(OH)D.

## Results

### Indexes comparison between different groups

In our study, we analyzed the association of 25(OH) D with UA in 9220 Chinese adults, including 3681 males (age 55.57 ± 13.23 years) and 5539 females (age 54.31 ± 12.83 years). Levels of SUA levels were 352.07 ± 79.25 nmol/L and 269.29 ± 64.68 nmol/L in males and females, respectively. The proportion of HUA was 12.26%. Blood glucose (FPG, HbA1c), lipids (TC, TG, LDL) and blood pressure (SBP, DBP) in HUA were higher than in the normal population. The HUA group also had a higher BMI and WC than did the normal group. There was also a significant difference in 25(OH) D in these two groups (40.28 ± 12.97 vs. 42.22 ± 13.23, *P* < 0.001) (Table 1).

**Table 1:** Baseline characteristics between different groups

Variables	Normal UA (*n* = 8090)	HUA (*n* = 1130)	*P* value
Age (years)	54.56 ± 12.94	56.62 ± 13.31	< 0.001
Male%	37.4	58.2	< 0.001
FPG (mmol/L)	5.57 ± 1.50	5.73 ± 1.34	0.001
HbA1c (%)	5.68 ± 0.99	5.79 ± 0.94	0.001
Fasting insulin (mIU/L)	42.30 ± 47.86	54.67 ± 62.35	< 0.001
HOMA-IR	1.59 ± 2.91	2.15 ± 4.04	< 0.001
ALT (IU/L)	20.75 ± 15.44	28.06 ± 20.61	< 0.001
TG (mmol/L)	1.58 ± 1.26	2.37 ± 2.39	< 0.001
TC (mmol/L)	5.19 ± 1.14	5.44 ± 1.10	< 0.001
LDL (mmol/L)	3.25 ± 0.81	3.43 ± 0.82	< 0.001
25(OH) D (nmol/L)	40.28 ± 12.97	42.22 ± 13.23	< 0.001
BMI (kg/m^2^)	24.46 ± 3.56	26.33 ± 3.45	< 0.001
WC (cm)	80.80 ± 10.23	87.12 ± 9.25	< 0.001
SBP (mmHg)	131.76 ± 21.74	137.00 ± 20.74	< 0.001
DBP (mmHg)	79.03 ± 13.02	82.94 ± 13.13	< 0.001
Diabetes%	14.5%	20.3%	< 0.001
Hypertension%	45.9%	61.5%	< 0.001

Data are presented as the mean ± standard deviation for continuous variables or as a number with proportion for categorical variables

*FPG* Fasting plasma glucose, *ALT* Alanine aminotransferase, *TG* Triglycerides, *TC* Total cholesterol, *BMI* Body mass index, *WC* Waist circumference, *SBP* Systolic blood pressure, *DBP* Diastolic blood pressure

### Comparison between different UA subgroups

UA was categorized into quartiles, and different indexes were compared between quartiles. Subjects with higher UA displayed increased blood glucose (FPG, HbA1c), lipids (TC, TG, LDL) and blood pressure (SBP, DBP). Other parameters, such as 25(OH) D, ALT, WC and BMI, showed graded changes as UA increased (*P* < 0.001) (Table 2).

**Table 2:** Comparison between different UA subgroups

	UA				
	Q1(45–242)	Q2(243–293)	Q3(294–352)	Q4(353–752)	*P* for trend
Age (years)	51.92 ± 12.68	55.00 ± 12.68	56.60 ± 12.65	55.77 ± 13.51	< 0.001
FPG (mmol/L)	5.47 ± 1.58	5.60 ± 1.52	5.63 ± 1.49	5.65 ± 1.29	< 0.001
HbA1c (%)	5.60 ± 1.10	5.71 ± 1.01	5.73 ± 0.93	5.73 ± 0.89	< 0.001
Fasting insulin (mIU/L)	37.34 ± 29.50	41.39 ± 38.96	45.61 ± 51.03	50.79 ± 69.64	< 0.001
HOMA-IR	1.37 ± 1.87	1.56 ± 2.49	1.73 ± 2.97	1.97 ± 4.36	< 0.001
ALT (IU/L)	17.46 ± 10.75	20.20 ± 17.18	22.06 ± 15.29	26.91 ± 19.41	< 0.001
TG (mmol/L)	1.30 ± 0.95	1.52 ± 1.10	1.73 ± 1.47	2.16 ± 2.00	< 0.001
TC (mmol/L)	5.09 ± 1.34	5.22 ± 1.05	5.26 ± 1.05	5.31 ± 1.08	< 0.001
LDL (mmol/L)	3.12 ± 0.79	3.29 ± 0.81	3.33 ± 0.80	3.36 ± 0.82	< 0.001
25(OH) D (nmol/L)	38.86 ± 12.19	40.07 ± 13.22	41.06 ± 13.27	42.11 ± 13.14	< 0.001
BMI (nmol/L)	23.54 ± 3.29	24.35 ± 3.70	24.97 ± 3.54	25.92 ± 3.44	< 0.001
WC (cm)	76.70 ± 9.42	79.96 ± 10.21	83.14 ± 9.60	86.65 ± 9.29	< 0.001
SBP (mmHg)	128.31 ± 21.72	132.01 ± 22.50	133.94 ± 21.06	135.40 ± 20.76	< 0.001
DBP (mmHg)	76.71 ± 12.75	78.54 ± 13.28	80.60 ± 12.70	82.26 ± 12.97	< 0.001

### Correlation of UA with other parameters

SUA was positively associated with 25(OH) D (*r* = 0.095, *P* < 0.001). Moreover, it also had a positive correlation with blood glucose (FPG, HbA1c), lipids (TC, TG, LDL) and blood pressure (SBP, DBP). HOMAIR, ALT BMI and WC were also associated with UA in our cohort (Table 3).

**Table 3:** Pearson correlation of UA with other parameters

	UA	
	*r*	*P*
Age (years)	0.101	< 0.001
FPG (mmol/L)	0.041	< 0.001
HbA1c (%)	0.043	< 0.001
Fasting insulin (mIU/L)	0.101	< 0.001
HOMA-IR	0.072	< 0.001
ALT (IU/L)	0.223	< 0.001
TG (mmol/L)	0.244	< 0.001
TC (mmol/L)	0.073	< 0.001
LDL (mmol/L)	0.100	< 0.001
25(OH) D (nmol/L)	0.095	< 0.001
BMI (kg/m^2^)	0.253	< 0.001
WC (cm)	0.370	< 0.001
SBP (mmHg)	0.126	< 0.001
DBP (mmHg)	0.173	0.000

### Liner regression analysis of UA and 25(OH)D

After adjusting for gender, age, ALT, TG, SBP, HbA1c and BMI, SUA concentration was significantly associated with 25(OH) D, with an unstandardized coefficient of 0.19 (95%CI 0.08, 0.30). However, SBP did not have a significant association with SUA (*P* = 0.151) (Table 4).

**Table 4:** Association between UA (dependent variable) and potential predictors (independent variables) by linear regression

	B	95%CI	*P*
25OHD	0.19	0.08, 0.30	0.001
Gender	−73.67	−76.61, −70.72	< 0.001
Age	0.47	0.35, 0.59	< 0.001
ALT	0.46	0.37, 0.55	< 0.001
TG	8.51	7.53, 9.49	< 0.001
SBP	0.05	−0.02, 0.13	0.151
HbA1c	−6.24	−7.75, −4.74	< 0.001
BMI	3.62	3.20, 4.04	< 0.001

Data are expressed as unStandardized B (95%CI). The enter procedure was used

### Logistic regression analysis of UA

It was found that 25(OH) D, age, ALT, TG and BMI were positively associated with UA. Gender and HbA1c were negatively associated with UA. After adjustment were made for gender, age, ALT, TG, SBP, HbA1c and BMI, the incidence of HUA was increased 9.4% for every 10-nmol/L increase in 25(OH) D (*P* < 0.001) (Table 5).

**Table 5:** Association between UA (dependent variable) and potential predictors (independent variables) by logistic regression

	Exp(B)	95%CI	*P*
25(OH) D (binned)	1.094	1.04, 1.15	< 0.001
Gender	0.53	0.46, 0.61	< 0.001
Age	1.01	1.01, 1.02	< 0.001
ALT	1.01	1.01, 1.02	< 0.001
TG	1.22	1.17, 1.28	< 0.001
SBP	1.00	1.00, 1.01	0.044
HbA1c	0.914	0.85, 0.98	0.013
BMI	1.10	1.08, 1.12	< 0.001

Data are expressed as Exp(B) (95%CI)

## Discussion

We performed a cross-sectional study in the general population. The level of 25(OH) D was higher in hyperuricemic than in normouricemic subjects. Furthermore, 25(OH) D was positively associated with SUA, even after adjustments were made for different variants. The incidence of HUA increased 9.4% for every 10-nmol/L increase in 25(OH)D.

The conclusions of some other studies were similar to ours. Sipahi S et al. found that a decrease in SUA was among the predictors of hypovitaminosis D [14]. However, several previous studies have concluded that HUA is associated with hypovitaminosis D [13, 15, 16]. This finding seems to indicate a complicated relationship between vitamin D status and SUA.

Vitamin D produced in the skin or obtained from the diet should undergo two steps of metabolic activation to become the active hormone (1,25(OH)_2_D). The first step, which results in 25-hydroxylated vitamin D, is conducted mostly in the liver by hydroxylases. In the circulation, 25(OH) D is bound to vitamin D-binding protein (DBP). The next hydroxylation occurs after the complexes of 25(OH) D and DBP are reabsorbed from the glomerular filtrate at the proximal tubule of the kidney. The production of 1,25(OH)_2_D is regulated by specific hormones on the expression of CYP27B1 and CYP24A1. CYP27B1 activates vitamin D metabolites, while CYP24 A1(24-hydroxylase enzyme) inactivates both 25(OH) D and 1,25(OH)_2_D, thus maintaining calcium and phosphate homeostasis [2]. The effect of vitamin D is far more extensive. The nonskeletal effects indicated that vitamin D was involved in a wide variety of pathologic processes. Some studies have reported that plasma 25(OH) D is associated with metabolic syndrome [17]. Additionally, vitamin D controls multiple biological processes, such as the following: cellular growth; angiogenesis or even modulation of the immune [18] and cardiovascular system [19], differentiation of keratinocytes [1]; and inhibition of the proliferation of breast [20], colon [21] and prostate cancer cells [22].

A high level of UA is considered to be associated with impaired renal function [23] and gouty arthritis [24]. Additionally, HUA may increase the risk of some diseases, such as CVD [25] or insulin resistance [26]. On the other hand, UA is a strong antioxidant. Nabipour I et al. found that a high level of UA was positively associated with higher bone mineral density (BMD) at all skeletal sites, serum calcium and 25(OH) D, as well as a lower prevalence of fractures in older men [27]. It is hypothesized that when liver function is impaired, both the production of UA and 25(OH) D decreases, because UA is produced in hepatocytes by xanthine oxidase, and vitamin D is hydroxylated in the liver to become 25(OH)D.

Estrogen may have influences on vitamin D and SUA [28, 29]. There was a different relationship between vitamin D and SUA in premenopausal women and postmenopausal women [13]. Vitamin D insufficiency was significantly associated with elevated UA among postmenopausal Chinese Han women, but no significant association was found among premenopausal women. It has been hypothesized that estradiol (E2) may affect SUA through mechanisms involving renal clearance, secretion and reabsorption [30]. Our study population included men, premenopausal women and postmenopausal women. We adjusted for gender and age, but we did not adjust for menopausal status, so the effect of estradiol may be confounded.

In addition, other factors may affect the SUA and 25(OH) D levels, such as sun exposure, vitamin D supplementation, and the use of certain drugs. Elevated parathyroid hormone (PTH) levels are thought to reduce renal urate excretion, although the exact mechanism remains unclear [31]. It was found that teriparatide therapy increased incidence of HUA in postmenopausal women [32]. SUA level returned to the pretreatment level after stopping treatment of PTH [33]. On the other hand, PTH can induce the expression of CYP27B1 and inhibit CYP24A1, as a result, the production of 1,25(OH)_2_D increases [2]. Therefore, hyperparathyroidism or PTH replacement can influence both SUA and vitamin D. We excluded the subjects who took anti-osteoporosis drugs, thus, no participant used PTH replacement.

UA is initially filtered in the kidney. Acute renal failure is associated with increased circulating SUA concentration as a decrease of renal excretion [10]. Reduced nephron mass and/or 1α-hydroxylase enzyme activity has been shown to be associated with a decline in 1, 25(OH)_2_D levels in patients with CKD [11]. As the substrate of 1,25(OH)_2_D, levels of 25(OH) D might be increased. Chen W et al. found that hyperuricemia suppresses 1α-hydroxylase, leading to lower 1,25(OH)_2_D and higher PTH in rats [30]. However, vitamin D is converted to 25(OH) D in the liver by 25-hydroxylase. Some studies have shown that treatment of HUA increases 1,25(OH)_2_D levels with no change in 25(OH)D [16, 34, 35]. In our study, we only measured the serum level of 25(OH) D to reflect vitamin D status. Thus, impaired renal function might raise SUA and 25(OH) D levels simultaneously.

Osteoporosis is a common public health problem in China. The prevalence of osteoporosis in China has increased over the past years, affecting more than one-third of people aged 50 years and older [36]. The most common prevention and treatment of osteoporosis is vitamin D supplementation. Although the causality between SUA and vitamin D was not clear, we should pay attention to the risk of hyperuricemia induced by excessive vitamin D supplements. More clinical trials are necessary to investigate the effect of vitamin D supplementation on serum UA.

Our study has several limitations that must be considered. First, we did not consider seasonal variation in 25(OH) D concentrations. Second, data on sun exposure were not available. Third, we did not measure serum calcium and parathyroid hormone, and we could not determine whether the association of 25(OH) D with SUA was partly mediated by calcium or secondary hyperparathyroidism, although individuals using anti-osteoporosis drugs were excluded. Fourth, the exact type and dose of alcohol were not available. In our questionnaire, we only recorded drinking status as never drinking and past or current drinking. In addition, diet-related data were not available in our study, so the influence of diet on SUA levels was not considered.

## Conclusion

Our findings in the eastern China population revealed that serum UA was positively associated with 25(OH) D, and the incidence of HUA increased 9.4% for every 10-nmol/L increase in 25(OH)D. Higher levels of serum 25(OH) D may be a potential predictor of HUA.

## Data Availability

The datasets used and/or analyzed during the current study are available from the corresponding authors on reasonable request.

## Abbreviations

UA: Uric acid
CVD: Cardiovascular disease
SUA: Serum uric acid
FPG: Fasting plasma glucose
WC: Waist circumference
HUA: Hyperuricemia
VDRs: Vitamin D receptors
NAFLD: Nonalcoholic fatty liver disease
CDK: Chronic kidney disease
HOMA-IR: Homeostasis model assessment of insulin resistance
BMI: Body mass index
SBP: Systolic blood pressure
DBP: Diastolic blood pressure
TC: Total cholesterol
TG: Triglycerides
LDL: Low-density lipoprotein
ALT: Alanine aminotransferase
BMD: Bone mineral density
PTH: Parathyroid hormone
